# 2-(2-{[2-(4-Pyridylcarbon­yl)hydrazinyl­idene]meth­yl}phen­oxy)acetic acid

**DOI:** 10.1107/S1600536810017083

**Published:** 2010-05-15

**Authors:** Jin Hong Xia, Bao Yu Liu, Zheng Liu

**Affiliations:** a College of Electronic Engineering (Guilin University of Electronic Technology), Guilin 541004, People’s Republic of China; b College of Chemical and Biological Engineering (Guilin University of Technology), Guilin 541004, People’s Republic of China

## Abstract

In the title compound, C_15_H_13_N_3_O_4_, the pyridine and benzene rings are nearly perpendicular [dihedral angle = 84.24 (5)°]. In the crystal structure, classical O—H⋯N hydrogen bonding between the OH group of the carboxyl unit and a neighbouring pyridine ring N atom and N—H⋯O hydrogen bonding between the imine NH group and a neighbouring O atom of an acyl unit, together with complementary non-classical C—H⋯O hydrogen bonds between carboxyl O atoms and neighbouring CH groups, link the mol­ecules into a three-dimensional system.

## Related literature

For hydrazones as corrosion inhibitors for metals and alloys, see: Fouda *et al.* (2000 ▶; 2007 ▶). For related structures, see: Chen *et al.* (2006 ▶); Hu *et al.* (2006 ▶).
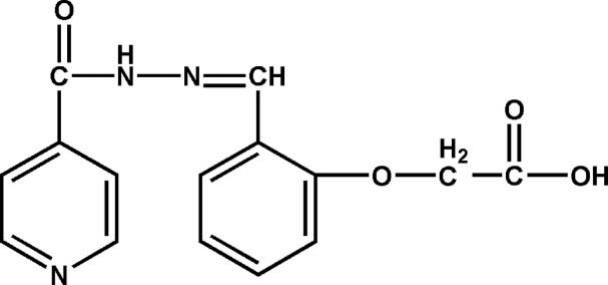


## Experimental

### 

#### Crystal data

C_15_H_13_N_3_O_4_
*M* *_r_* = 299.28
Orthorhombic, 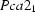

*a* = 12.8099 (12) Å
*b* = 4.9435 (5) Å
*c* = 21.921 (2) Å
*V* = 1388.2 (2) Å^3^
*Z* = 4
Mo *K*α radiation
μ = 0.11 mm^−1^
*T* = 296 K
0.49 × 0.21 × 0.18 mm

#### Data collection

Bruker APEXII CCD diffractometer
Absorption correction: multi-scan (*SADABS*; Bruker, 1998 ▶) *T*_min_ = 0.950, *T*_max_ = 0.981
11436 measured reflections
3189 independent reflections
2891 reflections with *I* > 2σ(*I*)
*R*_int_ = 0.023

#### Refinement

*R*[*F* ^2^ > 2σ(*F* ^2^)] = 0.033
*wR*(*F* ^2^) = 0.071
*S* = 1.02
3189 reflections
200 parameters
1 restraint
H-atom parameters constrained
Δρ_max_ = 0.17 e Å^−3^
Δρ_min_ = −0.16 e Å^−3^

### 

Data collection: *APEX2* (Bruker, 2004 ▶); cell refinement: *SAINT* (Bruker, 2004 ▶); data reduction: *SAINT*; program(s) used to solve structure: *SHELXS97* (Sheldrick, 2008 ▶); program(s) used to refine structure: *SHELXL97* (Sheldrick, 2008 ▶); molecular graphics: *SHELXTL* (Sheldrick, 2008 ▶); software used to prepare material for publication: *SHELXTL*.

## Supplementary Material

Crystal structure: contains datablocks global, I. DOI: 10.1107/S1600536810017083/rk2197sup1.cif

Structure factors: contains datablocks I. DOI: 10.1107/S1600536810017083/rk2197Isup2.hkl

Additional supplementary materials:  crystallographic information; 3D view; checkCIF report

## Figures and Tables

**Table 1:** Hydrogen-bond geometry (Å, °)

*D*—H⋯*A*	*D*—H	H⋯*A*	*D*⋯*A*	*D*—H⋯*A*
N2—H2*A*⋯O1^i^	0.86	2.01	2.8599 (18)	168
O4—H4*A*⋯N1^ii^	0.82	1.86	2.6337 (19)	156
C1—H1⋯O3^iii^	0.93	2.51	3.199 (2)	131
C4—H4⋯O3^iv^	0.93	2.58	3.315 (2)	136
C11—H11⋯O4^v^	0.93	2.43	3.347 (2)	171

Symmetry codes: (i) 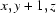; (ii) 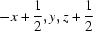; (iii) 
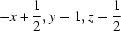
; (iv) 
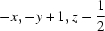
; (v) 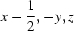.

**Fig. 1.**
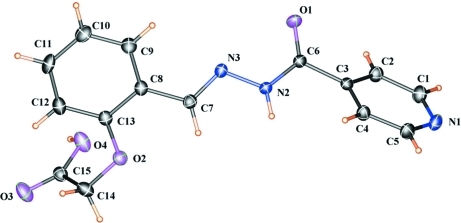
The molecular structure of title compound with the atom numbering scheme. Displacement ellipsoids are drawn at 30% probability level. H atoms are presented as a small spheres of arbitrary radius.

**Fig. 2.**
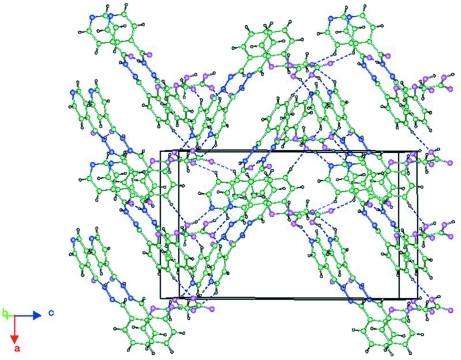
A view of the 3-dimensional system of hydrogen bonds.

## supplementary crystallographic information

### Comment

The hydrazone compounds have a strong ability of coordination, which have been investigated as corrosion inhibitors for metals and their alloys (Fouda *et al.*, 2000; 2007). The title compound (Fig.1) is closely related to the previously reported (*E*)-2-[2-(2,3-Dimethyl-5-oxo-1-phenyl-2,5-dihydro-1*H*-pyrazol-4- yliminomethyl) phenoxy]acetic acid monohydrate (Hu *et al.*, 2006) and 1-(4-Aminophenyl)ethanone isonicotinoylhydrazone (Chen *et al.*, 2006). The molecular structure of title compound reveals the nearly perpendicular system, in which dihedral angle between the pyridine and benzene rings is 84.24 (5)°. Adjacent molecules are connected by intermolecular classical O–H···N, N–H···O and non-classical C–H···O hydrogen bonds (Fig.2).

### Experimental

The methanol (10 ml) was added to an acetone solution (10 ml) of the 2-(2-{[2-(4-pyridylcarbonyl)hydrazono]methyl}phen-oxy)acetic acid (0.5 mmol). After stirring at 308 K for 2 h, crystals of the title compound were obtained by slow evaporation of the solution at room temperature.

### Refinement

The H atoms were placed in calculated positions (C–H = 0.93Å and 0.97Å, O–H = 0.82Å, N–H = 0.86Å) and were included in the refinement in the riding model approximation, with *U*_iso_(H) = 1.2*U*_eq_(C, N) and *U*_iso_(H) = 1.5*U*_eq_(O).

The 1548 Friedel pairs were merged in structure refinement procedure.

### Crystal data

**Table:**

C_15_H_13_N_3_O_4_	*F*(000) = 624
*M**_r_* = 299.28	*D*_x_ = 1.432 Mg m^−^^3^
Orthorhombic, *P**c**a*2_1_	Mo *K*α radiation, λ = 0.71073 Å
Hall symbol: P 2c -2ac	Cell parameters from 4508 reflections
*a* = 12.8099 (12) Å	θ = 3.2–27.8°
*b* = 4.9435 (5) Å	µ = 0.11 mm^−^^1^
*c* = 21.921 (2) Å	*T* = 296 K
*V* = 1388.2 (2) Å^3^	Block, yellow
*Z* = 4	0.49 × 0.21 × 0.18 mm

### Data collection

**Table:**

Bruker APEXII CCD diffractometer	3189 independent reflections
Radiation source: fine-focus sealed tube	2891 reflections with *I* > 2σ(*I*)
graphite	*R*_int_ = 0.023
φ and ω scans	θ_max_ = 27.5°, θ_min_ = 3.2°
Absorption correction: multi-scan (*SADABS*; Bruker, 1998)	*h* = −15→16
*T*_min_ = 0.950, *T*_max_ = 0.981	*k* = −6→6
11436 measured reflections	*l* = −28→28

### Refinement

**Table:**

Refinement on *F*^2^	Primary atom site location: structure-invariant direct methods
Least-squares matrix: full	Secondary atom site location: difference Fourier map
*R*[*F*^2^ > 2σ(*F*^2^)] = 0.033	Hydrogen site location: inferred from neighbouring sites
*wR*(*F*^2^) = 0.071	H-atom parameters constrained
*S* = 1.02	*w* = 1/[σ^2^(*F*_o_^2^) + (0.024*P*)^2^ + 0.395*P*] where *P* = (*F*_o_^2^ + 2*F*_c_^2^)/3
3189 reflections	(Δ/σ)_max_ < 0.001
200 parameters	Δρ_max_ = 0.17 e Å^−^^3^
1 restraint	Δρ_min_ = −0.15 e Å^−^^3^

### Special details

**Table:**

Geometry. All s.u.'s (except the s.u. in the dihedral angle between two l.s. planes) are estimated using the full covariance matrix. The cell s.u.'s are taken into account individually in the estimation of s.u.'s in distances, angles and torsion angles; correlations between s.u.'s in cell parameters are only used when they are defined by crystal symmetry. An approximate (isotropic) treatment of cell s.u.'s is used for estimating s.u.'s involving l.s. planes.
Refinement. Refinement of *F*^2^ against ALL reflections. The weighted *R*-factor w*R* and goodness of fit *S* are based on *F*^2^, conventional *R*-factors *R* are based on *F*, with *F* set to zero for negative *F*^2^. The threshold expression of *F*^2^ > σ(*F*^2^) is used only for calculating *R*-factors(gt) etc. and is not relevant to the choice of reflections for refinement. *R*-factors based on *F*^2^ are statistically about twice as large as those based on *F*, and *R*-factors based on ALL data will be even larger.

### Fractional atomic coordinates and isotropic or equivalent isotropic displacement parameters (Å^2^)

**Table:**

	*x*	*y*	*z*	*U*_iso_*/*U*_eq_	
C1	0.40315 (13)	−0.2335 (4)	0.17829 (9)	0.0418 (4)	
H1	0.4671	−0.3216	0.1815	0.050*	
C2	0.32247 (13)	−0.3168 (4)	0.21532 (8)	0.0367 (4)	
H2	0.3320	−0.4593	0.2424	0.044*	
C3	0.22713 (13)	−0.1862 (3)	0.21176 (7)	0.0296 (3)	
C4	0.21671 (14)	0.0228 (3)	0.16999 (7)	0.0362 (4)	
H4	0.1539	0.1154	0.1661	0.043*	
C5	0.30128 (15)	0.0903 (4)	0.13447 (8)	0.0435 (4)	
H5	0.2935	0.2295	0.1063	0.052*	
C6	0.13817 (12)	−0.2813 (3)	0.25116 (7)	0.0301 (3)	
C7	−0.04427 (13)	0.0500 (3)	0.33975 (7)	0.0315 (3)	
H7	−0.0125	0.2188	0.3371	0.038*	
C8	−0.13670 (12)	0.0137 (3)	0.37849 (7)	0.0298 (3)	
C9	−0.21215 (14)	−0.1804 (4)	0.36554 (8)	0.0389 (4)	
H9	−0.2033	−0.2925	0.3319	0.047*	
C10	−0.29905 (14)	−0.2103 (4)	0.40121 (9)	0.0424 (4)	
H10	−0.3485	−0.3415	0.3917	0.051*	
C11	−0.31293 (13)	−0.0451 (4)	0.45126 (9)	0.0452 (5)	
H11	−0.3725	−0.0636	0.4752	0.054*	
C12	−0.23880 (15)	0.1482 (4)	0.46622 (8)	0.0405 (4)	
H12	−0.2483	0.2577	0.5003	0.049*	
C13	−0.15039 (13)	0.1779 (3)	0.43020 (7)	0.0309 (3)	
C14	−0.08010 (16)	0.5251 (4)	0.49371 (8)	0.0418 (4)	
H14A	−0.1499	0.6013	0.4946	0.050*	
H14B	−0.0312	0.6739	0.4897	0.050*	
C15	−0.06001 (13)	0.3840 (3)	0.55373 (8)	0.0356 (4)	
N1	0.39396 (12)	−0.0327 (3)	0.13816 (7)	0.0420 (3)	
N2	0.07678 (10)	−0.0839 (3)	0.27315 (6)	0.0330 (3)	
H2A	0.0898	0.0824	0.2645	0.040*	
N3	−0.00745 (11)	−0.1488 (3)	0.30979 (6)	0.0340 (3)	
O1	0.12591 (11)	−0.5216 (2)	0.26232 (7)	0.0448 (3)	
O2	−0.07106 (9)	0.3570 (2)	0.44137 (5)	0.0368 (3)	
O3	−0.08545 (13)	0.4859 (3)	0.60119 (6)	0.0572 (4)	
O4	−0.01015 (11)	0.1540 (3)	0.54842 (6)	0.0478 (3)	
H4A	0.0110	0.1059	0.5820	0.072*	

### Atomic displacement parameters (Å^2^)

**Table:**

	*U*^11^	*U*^22^	*U*^33^	*U*^12^	*U*^13^	*U*^23^
C1	0.0328 (9)	0.0503 (10)	0.0421 (10)	0.0020 (8)	0.0033 (8)	0.0005 (9)
C2	0.0386 (9)	0.0369 (9)	0.0346 (8)	0.0012 (7)	0.0031 (7)	0.0057 (7)
C3	0.0344 (8)	0.0269 (7)	0.0275 (7)	−0.0027 (6)	0.0038 (6)	−0.0025 (6)
C4	0.0378 (9)	0.0334 (8)	0.0373 (9)	0.0048 (7)	0.0067 (7)	0.0048 (7)
C5	0.0541 (11)	0.0383 (9)	0.0381 (9)	−0.0008 (8)	0.0103 (9)	0.0073 (8)
C6	0.0323 (8)	0.0278 (8)	0.0301 (8)	−0.0019 (7)	0.0026 (7)	0.0007 (6)
C7	0.0332 (9)	0.0333 (8)	0.0280 (8)	−0.0021 (7)	−0.0003 (7)	0.0008 (7)
C8	0.0277 (8)	0.0350 (8)	0.0268 (7)	0.0037 (6)	−0.0011 (6)	0.0026 (7)
C9	0.0353 (9)	0.0464 (10)	0.0351 (9)	−0.0022 (8)	−0.0037 (7)	−0.0041 (8)
C10	0.0281 (8)	0.0508 (11)	0.0481 (10)	−0.0051 (8)	−0.0035 (8)	0.0056 (9)
C11	0.0283 (8)	0.0622 (12)	0.0451 (10)	0.0024 (8)	0.0101 (8)	0.0113 (9)
C12	0.0399 (10)	0.0475 (10)	0.0340 (8)	0.0093 (8)	0.0064 (8)	−0.0011 (8)
C13	0.0325 (8)	0.0323 (8)	0.0279 (8)	0.0052 (7)	−0.0015 (6)	0.0040 (7)
C14	0.0523 (11)	0.0339 (9)	0.0393 (9)	0.0047 (8)	−0.0032 (8)	−0.0066 (8)
C15	0.0337 (8)	0.0383 (8)	0.0348 (8)	−0.0010 (7)	−0.0024 (7)	−0.0064 (8)
N1	0.0413 (8)	0.0468 (9)	0.0380 (8)	−0.0091 (7)	0.0111 (7)	0.0012 (8)
N2	0.0370 (7)	0.0245 (6)	0.0374 (7)	−0.0034 (6)	0.0110 (6)	0.0001 (6)
N3	0.0345 (7)	0.0330 (7)	0.0344 (7)	−0.0016 (6)	0.0085 (6)	0.0017 (6)
O1	0.0527 (7)	0.0247 (6)	0.0571 (7)	−0.0024 (5)	0.0169 (6)	0.0047 (6)
O2	0.0426 (7)	0.0373 (6)	0.0304 (6)	−0.0024 (5)	0.0009 (5)	−0.0027 (5)
O3	0.0763 (10)	0.0575 (9)	0.0378 (7)	0.0115 (8)	0.0043 (7)	−0.0142 (7)
O4	0.0540 (8)	0.0562 (8)	0.0331 (6)	0.0224 (6)	−0.0052 (6)	−0.0021 (6)

### Geometric parameters (Å, °)

**Table:**

C1—N1	1.332 (2)	C9—C10	1.368 (2)
C1—C2	1.377 (2)	C9—H9	0.9300
C1—H1	0.9300	C10—C11	1.379 (3)
C2—C3	1.384 (2)	C10—H10	0.9300
C2—H2	0.9300	C11—C12	1.387 (3)
C3—C4	1.387 (2)	C11—H11	0.9300
C3—C6	1.505 (2)	C12—C13	1.388 (2)
C4—C5	1.375 (3)	C12—H12	0.9300
C4—H4	0.9300	C13—O2	1.3699 (19)
C5—N1	1.336 (2)	C14—O2	1.421 (2)
C5—H5	0.9300	C14—C15	1.511 (3)
C6—O1	1.2226 (19)	C14—H14A	0.9700
C6—N2	1.343 (2)	C14—H14B	0.9700
C7—N3	1.273 (2)	C15—O3	1.201 (2)
C7—C8	1.468 (2)	C15—O4	1.309 (2)
C7—H7	0.9300	N2—N3	1.3828 (18)
C8—C9	1.391 (2)	N2—H2A	0.8600
C8—C13	1.405 (2)	O4—H4A	0.8200
			
N1—C1—C2	123.09 (16)	C9—C10—H10	120.1
N1—C1—H1	118.5	C11—C10—H10	120.1
C2—C1—H1	118.5	C10—C11—C12	120.53 (16)
C1—C2—C3	119.32 (16)	C10—C11—H11	119.7
C1—C2—H2	120.3	C12—C11—H11	119.7
C3—C2—H2	120.3	C13—C12—C11	119.79 (16)
C2—C3—C4	118.02 (15)	C13—C12—H12	120.1
C2—C3—C6	119.35 (14)	C11—C12—H12	120.1
C4—C3—C6	122.59 (15)	O2—C13—C12	124.86 (15)
C5—C4—C3	118.59 (17)	O2—C13—C8	115.15 (13)
C5—C4—H4	120.7	C12—C13—C8	119.98 (15)
C3—C4—H4	120.7	O2—C14—C15	114.78 (14)
N1—C5—C4	123.72 (17)	O2—C14—H14A	108.6
N1—C5—H5	118.1	C15—C14—H14A	108.6
C4—C5—H5	118.1	O2—C14—H14B	108.6
O1—C6—N2	123.98 (15)	C15—C14—H14B	108.6
O1—C6—C3	121.04 (14)	H14A—C14—H14B	107.5
N2—C6—C3	114.97 (13)	O3—C15—O4	125.00 (18)
N3—C7—C8	120.21 (14)	O3—C15—C14	120.95 (16)
N3—C7—H7	119.9	O4—C15—C14	113.99 (15)
C8—C7—H7	119.9	C1—N1—C5	117.24 (15)
C9—C8—C13	118.44 (15)	C6—N2—N3	119.78 (13)
C9—C8—C7	121.77 (15)	C6—N2—H2A	120.1
C13—C8—C7	119.79 (14)	N3—N2—H2A	120.1
C10—C9—C8	121.54 (17)	C7—N3—N2	114.18 (13)
C10—C9—H9	119.2	C13—O2—C14	117.50 (14)
C8—C9—H9	119.2	C15—O4—H4A	109.5
C9—C10—C11	119.70 (17)		
			
N1—C1—C2—C3	−0.7 (3)	C11—C12—C13—O2	−178.35 (16)
C1—C2—C3—C4	0.8 (2)	C11—C12—C13—C8	0.5 (2)
C1—C2—C3—C6	178.57 (15)	C9—C8—C13—O2	177.56 (14)
C2—C3—C4—C5	−0.2 (2)	C7—C8—C13—O2	−2.4 (2)
C6—C3—C4—C5	−177.86 (16)	C9—C8—C13—C12	−1.4 (2)
C3—C4—C5—N1	−0.6 (3)	C7—C8—C13—C12	178.62 (15)
C2—C3—C6—O1	−36.1 (2)	O2—C14—C15—O3	−164.70 (17)
C4—C3—C6—O1	141.58 (18)	O2—C14—C15—O4	17.9 (2)
C2—C3—C6—N2	142.74 (15)	C2—C1—N1—C5	−0.1 (3)
C4—C3—C6—N2	−39.6 (2)	C4—C5—N1—C1	0.8 (3)
N3—C7—C8—C9	−28.5 (2)	O1—C6—N2—N3	−1.4 (3)
N3—C7—C8—C13	151.42 (15)	C3—C6—N2—N3	179.86 (13)
C13—C8—C9—C10	1.2 (3)	C8—C7—N3—N2	177.30 (14)
C7—C8—C9—C10	−178.87 (17)	C6—N2—N3—C7	163.73 (15)
C8—C9—C10—C11	0.0 (3)	C12—C13—O2—C14	−0.1 (2)
C9—C10—C11—C12	−1.0 (3)	C8—C13—O2—C14	−178.99 (14)
C10—C11—C12—C13	0.7 (3)	C15—C14—O2—C13	74.9 (2)

### Hydrogen-bond geometry (Å, °)

**Table:**

*D*—H···*A*	*D*—H	H···*A*	*D*···*A*	*D*—H···*A*
N2—H2A···O1^i^	0.86	2.01	2.8599 (18)	168
O4—H4A···N1^ii^	0.82	1.86	2.6337 (19)	156
C1—H1···O3^iii^	0.93	2.51	3.199 (2)	131
C4—H4···O3^iv^	0.93	2.58	3.315 (2)	136
C11—H11···O4^v^	0.93	2.43	3.347 (2)	171

Symmetry codes: (i) *x*, *y*+1, *z*; (ii) −*x*+1/2, *y*, *z*+1/2; (iii) −*x*+1/2, *y*−1, *z*−1/2; (iv) −*x*, −*y*+1, *z*−1/2; (v) *x*−1/2, −*y*, *z*.
